# CD4^+^ T Cells Are as Protective as CD8^+^ T Cells against *Rickettsia typhi* Infection by Activating Macrophage Bactericidal Activity

**DOI:** 10.1371/journal.pntd.0005089

**Published:** 2016-11-22

**Authors:** Kristin Moderzynski, Stefanie Papp, Jessica Rauch, Liza Heine, Svenja Kuehl, Ulricke Richardt, Bernhard Fleischer, Anke Osterloh

**Affiliations:** 1 Department of Immunology, Bernhard Nocht Institute for Tropical Medicine, Hamburg, Germany; 2 Institute for Immunology, University Medical Center Hamburg-Eppendorf, Hamburg, Germany; Naval Medical Research Center, UNITED STATES

## Abstract

*Rickettsia typhi* is an intracellular bacterium that causes endemic typhus, a febrile disease that can be fatal due to complications including pneumonia, hepatitis and meningoencephalitis, the latter being a regular outcome in T and B cell-deficient C57BL/6 RAG1^-/-^ mice upon *Rickettsia typhi* infection. Here, we show that CD4^+^ T_H_1 cells that are generated in C57BL/6 mice upon *R*. *typhi* infection are as protective as cytotoxic CD8^+^ T cells. CD4^+^- as well as CD8^+^-deficient C57BL/6 survived the infection without showing symptoms of disease at any point in time. Moreover, adoptively transferred CD8^+^ and CD4^+^ immune T cells entered the CNS of C57BL/6 RAG1^-/-^ mice with advanced infection and both eradicated the bacteria. However, immune CD4^+^ T cells protected only approximately 60% of the animals from death. They induced the expression of iNOS in infiltrating macrophages as well as in resident microglia in the CNS which can contribute to bacterial killing but also accelerate pathology. *In vitro* immune CD4^+^ T cells inhibited bacterial growth in infected macrophages which was in part mediated by the release of IFNγ. Collectively, our data demonstrate that CD4^+^ T cells are as protective as CD8^+^ T cells against *R*. *typhi*, provided that CD4^+^ T_H_1 effector cells are present in time to support bactericidal activity of phagocytes via the release of IFNγ and other factors. With regard to vaccination against TG *Rickettsiae*, our findings suggest that the induction of CD4^+^ T_H_1 effector cells is sufficient for protection.

## Author Summary

Endemic typhus caused by *Rickettsia typhi* usually is a relatively mild disease. However, CNS inflammation and neurological symptoms are complications that can occur in severe cases. This outcome of disease is regularly observed in T and B cell-deficient C57BL/6 RAG1^-/-^ mice upon infection with *R*. *typhi*. We show here that CD4^+^ T cells are as protective as cytotoxic CD8^+^ T cells against *R*. *typhi* as long as they are present in time. This is evidenced by the fact that neither CD8^+^ nor CD4^+^ T cell-deficient C57BL/6 mice develop disease which is also true for *R*. *typhi*-infected C57BL/6 RAG1^-/-^ mice that receive immune CD8^+^ or CD4^+^ at an early point in time. Moreover, adoptive transfer of immune CD4^+^ T cells still protects approximately 60% of C57BL/6 RAG1^-/-^ mice when applied later in advanced infection when the bacteria start to rise. Although CD8^+^ T cells are faster and more efficient in bacterial elimination, *R*. *typhi* is not detectable in CD4^+^ T cell recipients anymore. We further show that immune CD4^+^ T cells activate bactericidal functions of microglia and macrophages in the CNS *in vivo* and inhibit bacterial growth in infected macrophages *in vitro* which is in part mediated by the release of IFNγ. Collectively, we demonstrate for the first time that CD4^+^ T cells alone are sufficient to protect against *R*. *typhi* infection. With regard to vaccination our findings suggest that the induction of *R*. *typhi*-specific CD4^+^ T_H_1 effector T cells may be as effective as the much more difficult targeting of cytotoxic CD8^+^ T cells.

## Introduction

*Rickettsiae* (*R*.) are small obligate intracellular bacteria that are transmitted to humans by arthropod vectors. *R*. *prowazekii* and *R*. *typhi* represent the two members of the typhus group (TG) of *Rickettsiae* [1, 2] and are the causative agents of epidemic and endemic typhus, respectively. Both diseases appear with similar symptoms including high fever, headache, myalgia and joint pain, nausea and vomiting. Furthermore, neurological symptoms such as confusion and stupor are common [3]. Many patients develop a characteristic rash which is due to local blood vessel damage and inflammation as endothelial cells belong to the main target cells of these bacteria [4]. Fatal complications include pneumonia, myocarditis, nephritis and encephalitis/meningitis [3, 5] and are more common in epidemic typhus (20–30% lethality) [5–7]. The course of disease of endemic typhus caused by *R*. *typhi* is usually milder and the lethality is estimated to be <5% [7, 8] if untreated with antibiotics such as tetracyclins or chloramphenicol. As clinical presentations are often non-specific, endemic typhus, however, is clearly underdiagnosed and, thus, often unrecognized [3, 9].

Epidemic and endemic typhus generally occur worldwide. Epidemic typhus that is transmitted from human-to-human by the body louse sporadically appears in low-income countries of South America and Africa but also in upper-middle economies such as Peru [10] and Algeria [11] and industrial countries such as Russia [12]. The most recent larger outbreak of epidemic typhus was in the context of civil war in Burundi in 1995 [13]. Endemic typhus is much more prevalent and actually one of the most abundant rickettsial infections [14]. Rats and mice serve as natural reservoirs of *R*. *typhi* and the bacteria are transmitted to humans by fleas, predominantly the rat flea *Xenopsylla cheopis*, from these animals [14, 15]. Endemic areas are mainly found in low-income countries in Africa [16–22] and Asia [23–26] where the disease primarily occurs in warm coastal areas and ports where populations of rats and mice are numerous and hygienic standards are low. Within the past twenty years the incidence of endemic typhus has been increasing also in western countries. Rising case numbers are reported from the US (Hawaii [27] and Texas [28, 29]) as well as from Southern Europe (e.g. Cyprus [30, 31], Greece [32], Spain [33–36] including Canary Islands [37, 38]) and Portugal [39, 40]. Especially homeless people are at enhanced risk to acquire the infection. For example, 9.6% of the homeless were seropositive in Houston, Texas, in 2008 [41]. Another study indicates increasing occurrence of *R*. *typhi* in France. While 0.54% of the homeless in Marseille were seropositive in the years 2000–2003, seropositivity increased to 22% in the years 2010–2013 [42].

A vaccine against rickettsial infections is not available but clearly desired for several reasons. It is known that some rickettsial species persist and can re-appear. This is true for *R*. *prowazekii*, the closest relative of *R*. *typhi*. *R*. *prowazekii* can cause the so-called Brill-Zinsser disease years to decades after primary infection which appears with similar symptoms as the primary infection and is usually accompanied by meningitis and neurological symptoms [43–46]. Stress or waning immunity is suggested to re-activate *R*. *prowazekii* [47]. Similar may be true for *R*. *typhi* because we recently showed that *R*. *typhi* persists in mice [48]. Moreover, in mice it has been shown that *R*. *prowazekii* persists irrespective of antibiotic treatment [49]. In addition, there is the risk of the development of antibiotic resistances. Finally, TG *Rickettsiae* are considered potential bioweapons.

Vaccine development requires understanding of protective immune responses as well as of a possible contribution of immune reactions to pathology. To date little is known about immune response against *Rickettsiae* although animal models of rickettsial infections have been established. Current studies mainly focused on immunity against spotted fever group (SFG) *Rickettsiae* that represent the vast majority of *Rickettsiae* but phylogenetically differ from TG *Rickettsiae*. Especially immunity against *R*. *conorii* and *R*. *rickettsii* of this group has been studied in mice. Generally, BALB/c and C57BL/6 mice have been reported to be resistant against rickettsial infections [50–54] while C3H/HeN mice were found to be susceptible [50, 54]. In C3H/HeN mice it has been shown that CD8^+^ T cells that can directly kill infected cells play an important role in defense against *Rickettsiae*. CD8^+^ T cells showed enhanced cytotoxic activity in C3H/HeN mice upon infection with *R*. *conorii* and *R*. *australis* [55]. Furthermore, depletion of CD8^+^ T cells led to enhanced susceptibility of C3H/HeN mice to *R*. *conorii* and *R*. *australis* [55, 56] as well as to *R*. *typhi* [57] as reflected by enhanced bacterial burden and pathology while adoptive transfer of CD8^+^ immune T cells protected C3H/HeN mice against a lethal dose of *R*. *conorii* [56]. This was also true for adoptively transferred CD4^+^ T cells [56]. An important effector molecule produced by T cells as well as by NK cells is IFNγ. This cytokine has been shown to contribute to rickettsial control. C57BL/6 IFNγ^-/-^ mice succumbed to the infection with a normally sublethal dose of *R*. *conorii*. Furthermore, neutralization of IFNγ led to severe disease in C3H/HeN mice upon *R*. *conorii* infection [58]. Similar was also true for the neutralization of TNFα [58] which can be produced by various cell types including CD4^+^ T cells and macrophages (MΦ) [59, 60]. Nevertheless, C57BL/6 RAG1^-/-^ mice that lack adaptive immunity survive the infection with *R*. *conorii* at least for 20 days [61] which is also true for *R*. *typhi* [48], suggesting that innate immune mechanisms can control the bacteria at least for a certain period of time. In C57BL/6 RAG1^-/-^ mice, however, *R*. *typhi*, re-appears several months after infection and then grows predominantly in the brain [48]. Animals develop massive CNS inflammation accompanied by neuronal cell loss and succumb to neurological disorders [48]. These findings clearly demonstrate the need of adaptive immunity for the control of persisting *R*. *typhi*.

In the present study we show that CD4^+^ T cells are sufficient to protect against *R*. *typhi* in the C57BL/6 RAG1^-/-^ infection model by activating MΦ via IFNγ and other factors.

## Materials and Methods

### Ethics statement

All experimentations and procedures were approved by the Public Health Authorities (Amt für Gesundheit und Verbraucherschutz, Hamburg; No 61/12 and No 88/13) and performed according to the German Animal Welfare Act.

### Mice

C57BL/6, C57BL/6 RAG1^-/-^ [62], C57BL/6 MHCI^-/-^ lacking CD8^+^ cytolytic cells [63, 64] and C57BL/6 MHCII^-/-^ lacking CD4^+^ T cells [65, 66] mice were bred in the animal facilities of the Bernhard Nocht Institute for Tropical Medicine and housed in a biosafety level 3 facility for experimentation. The facilities are registered by the Public Health Authorities (Amt für Gesundheit und Verbraucherschutz, Hamburg).

### Culture and purification of *R*. *typhi*

*R*. *typhi* (strain Wilmington, accession no. AE017197) was cultured in L929 mouse fibroblasts (ATCC CCL-1) in RPMI1640 (PAA, Cölbe, Germany) supplemented with 10% FCS (PAA, Cölbe, Germany), 2 mM L-glutamine (PAA, Cölbe, Germany) and 10 mM HEPES (PAA, Cölbe, Germany) without antibiotics (standard culture medium). 1×10^7^ γ-irradiated (1966 rad) L929 cells were seeded in 175 cm^2^ culture flasks (Greiner Bio-One, Frickenhausen, Germany). One day later cells were infected with *R*. *typhi* and incubated for 5 to 7 days. For the preparation of bacterial stocks, infected L929 cells were resuspended in 1.5 ml PBS. 200 μl silicium particles (60/90 grit silicon carbide; Lortone inc., Mukilteo, USA) were added and cells were vortexed thoroughly for 1 min. The crude lysate was strained through a 2 μm cell strainer (Puradisc 25 syringe filter 2 μm; GE Healthcare Life Sciences, Freiburg, Germany). Bacteria were centrifuged at 4300×g for 5 min at room temperature and frozen in FCS with 7.5% DMSO in liquid nitrogen in Cryo.S tubes (Greiner Bio-One, Frickenhausen, Germany). Thawed bacterial stocks were centrifuged at 6200×g for 5 min at room temperature, washed twice with PBS and analyzed for bacterial content by quantitative real-time PCR (qPCR) and immunofocus assay as described previously [48] to determine spot forming units (sfu).

### Infection of mice

*R*. *typhi* stocks were thawed and washed in PBS as described above. 2×10^6^ sfu were administered in 50 μl PBS subcutaneously (s.c.) into the base of the tail. Blood samples were obtained by submandibular bleeding.

### Clinical scoring

The state of health was evaluated by a clinical score. The following parameters were assessed: body mechanics/motion (0: normal; 1: tremor/swaying motion; 2: paresis/ataxia; 3: paralysis) and weight loss (0: normal (<10%); 1: mild (>10%), 2: severe (>15%)) giving a maximum score of 5. Mice were considered healthy with a score ≤1, moderately ill with a score of 2–3 and severely ill with a score of 4–5. Mice were sacrificed reaching a total score of ≥4 or showing weight loss of >20%.

### DNA preparation from purified bacteria, cell cultures and organs

DNA was prepared from purified bacteria, cell cultures and organs employing the QIAamp DNA Mini Kit (QIAGEN, Hilden, Germany). 10 mg tissue were homogenized in 500 μl PBS in Precellys ceramic Kit tubes (Peqlab, Erlangen, Germany) in a Precellys 24 homogenizer (Peqlab, Erlangen, Germany; two times 6000 rpm for 45 sec with a 60 sec break). 80 μl tissue homogenizate or up to 1×10^6^ cells were used for DNA preparation according to the manufacturer´s instructions.

### qPCR

Quantification of purified *R*. *typhi*, bacteria in cell cultures and organs from infected mice was performed by amplification of a 137 bp fragment of the *PrsA* gene as previously described [48].

### Detection of cytokines by LegendPLEX assay

Cytokines were detected in cell culture supernatants with LegendPLEX assay (Biolegend, London, UK) according to the manufacturer´s instructions. Cell culture supernatants were diluted 1:2–1:10.

### Detection of nitric oxide (NO) by Griess reaction

50 μl Griess 1 reagent (0.5 g sulfonamide in 50 ml 1M HCl) and 50 μl Griess 2 reagent (0.15 g naphtylethylendiamine-dihydrochloride in 50 ml H_2_O) were added to 100 μl cell culture supernatant in microtiter plates (Greiner Bio-One, Frickenhausen, Germany). A serial dilution of sodium nitrite (NaNO_2_) in cell culture medium was used as a standard (c_max_ 125 μM). The absorbance was measured at 560 nm with a Dynex MRXII spectrophotometric microplate reader (Dynex Technologies, Chantilly, USA).

### Flow cytometry

Brain from naïve and infected mice were homogenized. In addition, blood samples were analyzed. Erythrocyte lysis was performed for blood by incubating the cells in erythrocyte lysis buffer (10 mM Tris, 144 mM NH_4_Cl, pH7.5) for 5 min at RT. Cells were afterwards washed two times in PBS. Brain and spinal cord cells were strained through a 30 μm CellTrics cell strainer (Partec, Görlitz, Germany) and directly used for stainings. Cells were fixed and permeabilized with Cytofix/Cytoperm and Perm/Wash solutions (BD Biosciences, Heidelberg, Germany) according to the manufacturer´s protocol. Fc receptors were blocked with 5% CohnII human IgG fraction (Sigma-Aldrich, Deisenhofen, Germany) in Perm/Wash for 15 min at 4°C followed by the addition of either mouse anti-*R*. *typhi* (BNI52) [48] or mouse IgG3 isotype antibody (clone B10; SouthernBiotech, Birmingham, USA), each at a concentration of 1 μg/ml in Perm/Wash. Cells were washed in Perm/Wash after 20 min of incubation at 4°C followed by incubation for 20 min at 4°C with rat anti-mouse IgG3-FITC (1:200 in Perm/Wash; #1100–02, SouthernBiotech, Birmingham, USA). After washing cells were further stained with rat anti-mouse iNOS-PE (clone CXNFT; eBioscience, Frankfurt, Germany) and rat anti-mouse CD11b-PerCPCy5.5. (clone M1/70; BD Biosciences, Heidelberg, Germany). To discriminate immune cells in brain, cells were additionally stained with rat anti-mouse CD45-AF647 (clone 30-F11; Biolegend, London, UK). Rat anti-iNOS-PE, rat anti-CD11b-PerCPCy5.5. and rat anti-mouse CD45-AF647 were used at 1:200 dilutions in Perm/Wash solution. After 20 min at 4°C cells were finally washed and resuspended in PBS/1% paraformaldehyde. CD4^+^ and CD8^+^ T cells were stained extracellularly in blood with anti-mouse CD4-PE (clone GK1.5; BD Biosciences, Heidelberg, Germany; 1:200) and anti-mouse CD8-PerCP-Cy5.5 (clone 53–6.7; BD Biosciences, Heidelberg, Germany; 1:200) and in brain with anti-mouse CD4 PerCPCy5.5 (clone RM4-5; eBioscience, Frankfurt, Germany; 1:200) and anti-mouse CD8-Alexa488 (clone 53–6.7; Biolegend, London, UK; 1:200). In the brain, cells were additionally stained with rat anti-mouse CD45-AF647 (clone 30-F11; Biolegend, London, UK) to identify CD45^high^ infiltrating cells. KLRG1 and CD11a were detected by extracellular staining employing anti-mouse KLRG1-PE (clone 2F1/KLRG1; Biolegend, London, UK; 1:800) and anti-mouse CD11a-eFluor450 (clone M17/4; eBioscience, Frankfurt, Germany; 1:200). Intracellular IFNγ and Granzyme B were detected with anti-mouse IFNγ-PE/Dazzle (clone XMG1.2; Biolegend, London, UK; 1:333) and anti-mouse Granzyme B-PacificBlue (clone GB11, Biolegend, London, UK; 1:200) in spleen cells restimulated with 10 ng/ml PMA and 500 ng/ml Ionomycin in 200 μl in 96well plates in the presence of 1 μl GolgiStop (BD Biosciences, Heidelberg, Germany) for 4h and permeabilized with cytofix/cytoperm (BD Biosciences, Heidelberg, Germany) according to the manufacturer´s protocol. For the analysis of surface expression of MHC molecules and CD80 bmMΦ were stained with anti-mouse CD11b-BV421 (clone M1/70, Biolegend, London, UK; 1:50). *R*. *typhi* was detected *by* intracellular staining with anti-*R*. *typhi* BNI52 (1 μg/ml), anti-mouse IgG3-FITC (#1100–02, SouthernBiotech, Birmingham, USA; 1:200) and anti-FITC-Alexa488 (Thermo Fisher Scientific, Braunschweig, Germany; 1:1000). MHC molecules and CD80 were stained extracellularly with anti-mouse MHCI (H2-K^b^)-PE (AF6-88.5.5.3, eBioscience, Frankfurt, Germany; 1:50), anti-mouse MHCII (I-A/I-E)-APC (M5/114.15.2, eBioscience, Frankfurt, Germany; 1:50) and anti-mouse CD80-PE/Dazzle (16-10A1, Biolegend, London, UK; 1:50). Analysis was performed employing a Accuri C6 (BD Biosciences, Heidelberg, Germany) or LSRII flow cytometer (BD Biosciences, Heidelberg, Germany) and FlowJo software (FlowJo LLC, Ashland, USA).

### Generation of bone marrow-derived MΦ (bmMΦ)

bmMΦ were generated from tibia and femur of the hind legs. 2×10^6^ bone marrow cells were cultured in petri dishes (Sarstedt, Nuembrecht, Germany) in IMDM medium (PAA Laboratories, Cölbe, Germany) supplemented with 2 mM L-glutamine, 5% horse serum and 10% M-CSF-containing cell culture supernatant from L929 fibroblasts. Medium was exchanged every 3 days. Cells were harvested for experimentation after 10–12 days of culture. Virtually 100% of the cells were CD11b^+^F4/80^+^ MΦ.

### Infection of bmMΦ with *R*. *typhi in vitro* for the assessment of cytokine and NO production, MHCI, MHCII and CD80 expression and bacterial growth

For the analysis of cytokine and NO production and the expression of MHCI, MHCII and CD80 on the cell surface 2×10^5^ bmMΦ were infected with 10, 25 or 50 copies of *R*. *typhi* per cell in 24well plates. Control cells were left untreated or stimulated with LPS (500 ng/ml). Supernatants and cells were harvested at 24h and 48h post infection. Bacterial content and the expression of MHCI, MHCII and CD80 on the cell surface was analyzed by flow cytometry at 24h and 48h. Cytokines and NO were detected in the supernatant of cultures that were infected with 50 copies *R*. *typhi*/cell at 48h by LegendPLEX assay and Griess reaction. For the analysis of bacterial growth 2×10^5^ bmMΦ were infected with 5 copies *R*. *typhi* per cell. Free bacteria were washed out 3h afterwards and cells were further incubated for 96h. At indicated points in time qPCR was performed to quantify *R*. *typhi*.

### Isolation of CD4^+^ and CD8^+^ T cells and adoptive transfer into *R*. *typhi*-infected C57BL/6 RAG1^-/-^ mice

Immune CD4^+^ and CD8^+^ T cells were isolated from C57BL/6 mice on day 21 post *R*. *typhi* infection employing the MagniSort Mouse CD4 and CD8 T cell enrichment kits from eBioscience (Frankfurt, Germany) using an EasySep Magnet from Stemcell Technologies (Köln, Germany). Procedures were performed according to the manufacturer´s instructions. Purity was analyzed by flow cytometry and found to be >98% for each cell population in all preparations. CD4^+^ T cells were generally absent in CD8^+^ T cell preparations and vice versa. 1×10^6^ CD4^+^ or CD8^+^ T cells were injected i.v. in 100 μl PBS into C57BL/6 RAG1^-/-^ mice 63 days after *R*. *typhi* infection, which is the point in time when bacteria become detectable by qPCR in the brain and approximately 20–40 days prior to the usual onset of disease. *R*. *typhi*-infected control animals received PBS instead of T cells. Additional C57BL/6 RAG1^-/-^ control mice were not infected but received either CD4^+^ or CD8^+^ immune T cells.

### T cell activation and bacterial killing by bmMΦ *in vitro*

1×10^6^ bmMΦ were infected with 5 copies *R*. *typhi* per cell in 24well plates. Free bacteria were washed out after 3h and cells were further incubated for 24h at 37°C to allow bacterial entry and replication. Immune CD4^+^ T cells were isolated as described above from *R*. *typhi*-infected C57BL/6 wild-type mice 7 days post infection. Control CD4^+^ T cells were purified from the spleen of mice that received PBS instead. 1.5×10^6^ purified CD4^+^ T cells were added per well and cells were further incubated for 72h. IFNγ was neutralized by simultaneous addition of anti-IFNγ (1 μg/ml; BioXCell, West Lebanon, USA). In another experimental setup 1×10^6^ bmMΦ were infected with 5 copies *R*. *typhi* per cell. After 3h hours the medium was exchanged by cell culture medium with or without recombinant IFNγ (10 U/ml; Merck Millipore, Eschborn, Germany) or a combination of recombinant IFNγ that was pre-incubated with anti-IFNγ (1 μg/ml) for 15 min at RT for neutralization. Supernatants and cells were harvested 96h after infection. Cytokines and NO were quantified in the supernatants by LegendPLEX and Griess assay. Bacterial content was determined in the cell pellet by *PrsA* qPCR.

### Histological stainings

For immunohistochemistry (IHC) tissues from infected mice were fixed in 4% formalin in PBS and embedded in paraffin. Deparaffinization of the sections was performed using standard methods. Sections were first heated at 63°C for 30 min in a heating cabinet followed by treatment with Xylol for 30 min and EtOH (3x 100% EtOH, 3x 96% EtOH, 80% EtOH, 70% EtOH). Each step was performed for 3–5 min. Slides were finally washed in H_2_O. Deparaffinized sections were boiled for 30 min in 10 mM citrate buffer (10 mM sodium citrate, 0.05% Tween20, pH6.0) for antigen retrieval. Staining was performed using a Ventana Benchmark XT apparatus (Ventana, Tuscon, USA). Antibodies were diluted in 5% goat serum (Dianova, Hamburg, Germany) in Tris-buffered saline pH7.6 (TBS) and 0.1% Triton X100 in antibody diluent solution (Zytomed, Berlin, Germany). Rabbit anti-mouse CD3 (1:100; clone SP7; Abcam, Cambridge, USA), rabbit anti-mouse IBA1 (1:500; #019–19741; WAKO, Neuss, Germany) and rabbit anti-mouse iNOS (1:75; ABIN373696, Abcam, Cambridge, USA) were used. Anti-rabbit or anti-rat Histofine Simple Stain Mouse MAX peroxidase-coupled antibodies (Nichirei Biosciences, Tokyo, Japan) were used as secondary antibodies. Detection was performed with ultraview universal DAB detection kit (Ventana, Tuscon, USA). Sections were covered with Tissue-Tek embedding medium (Sakura Finetek, Staufen, Germany). Images were taken with a BZ9000 Keyence microscope (Keyence, Neu-Isenburg, Germany).

### Statistical analysis

Statistical analysis was performed with GraphPad Prism 5 software (GraphPad Software Inc., La Jolla, USA). Student´s T test, Mann-Whitney U test, or One-way ANOVA test followed by Kruskal-Wallis and Dunn´s post test were performed as indicated in the figure legends.

## Results

### C57BL/6 mice mount a cytotoxic CD8^+^ T cell and a CD4^+^ T_H_1 response in *R*. *typhi* infection

**Fig 1:**
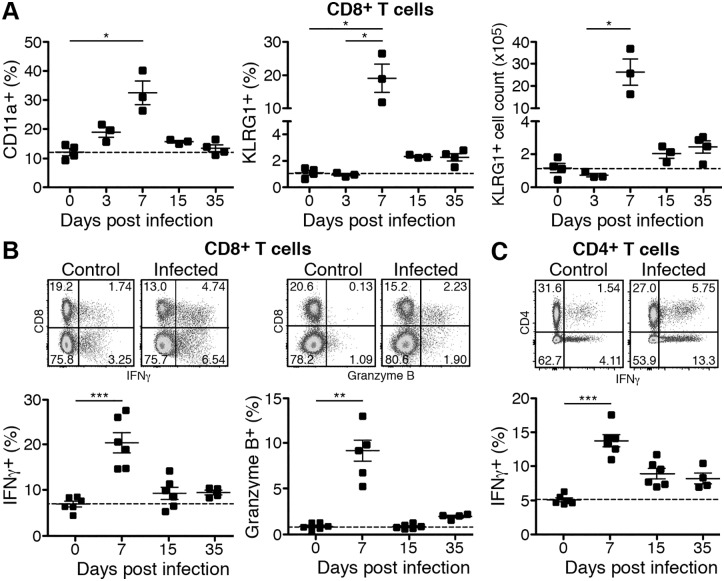
C57BL/6 mice mount a cytotoxic CD8^+^ T cell and CD4^+^ T_H_1 response. CD8^+^ T cells in the spleen from non-infected control mice (d0) and *R*. *typhi*-infected C57BL/6 mice were analyzed for CD11a and KLRG1 expression at indicated points in time (x-axis). The percentage of CD11a^+^ and KLRG1^+^ as well as the absolute numbers of KLRG1-expressing CD8^+^ T cells (y-axis) are depicted (**A**). Spleen cells were restimulated *in vitro* with PMA and ionomycin. CD8^+^ T cells were analyzed for intracellular expression of IFNγ and granzyme B by flow cytometry. Representative dot plots of the stainings from a control mouse and a *R*. *typhi*-infected mouse at day 7 post infection are depicted. Graphs show the percentage of IFNγ^+^ and Granzyme B^+^ cells among CD8^+^ T cells (y-axis) at indicated points in time (x-axis) (**B**). PMA/Ionomycin-restimulated CD4^+^ T cells from the same cultures were analyzed for intracellular expression of IFNγ by flow cytometry. Representative dot plots of the stainings from a control mouse and a *R*. *typhi*-infected mouse at day 7 post infection are depicted. Graphs show the percentage of IFNγ^+^ cells among CD4^+^ T cells (y-axis) at indicated points in time (x-axis) (**C**). Each symbol represents a single mouse. Statistical analysis was performed with Kruskal-Wallis and Dunn´s post test. Asterisks indicate statistically significant differences compared to day 0 (*p<0.05, **p<0.01, ***p<0.001).

T cells, especially cytotoxic CD8^+^ T cells, are important in the elimination of intracellular pathogens. Therefore, we first analyzed the CD8^+^ T cell response in *R*. *typhi*-infected C57BL/6 mice during the course of infection. For these analyses spleen cells were re-stimulated *in vitro* with PMA/Ionomycin followed by flow cytometric analysis of IFNγ expression and the expression of Granzyme B as a cytotoxic effector molecule. In addition, CD8^+^ T cells from the spleen of the mice were analyzed for the expression of KLRG1 as a marker for terminally differentiated non-replicative T cells [67, 68] and CD11a as a marker for antigen-experienced cells [69]. Analyses were performed on days 0 (naïve), 3, 7, 15 and 35 post infection. CD8^+^CD11a^+^ T cells began to rise on day 3, peaked on day 7 and declined until day 15 (Fig 1A, left). Differentiated CD8^+^KLRG1^+^ T cells peaked on day 7 and were not yet detectable on day 3 post infection (Fig 1, middle). CD8^+^KLRG1^+^ T cells also declined until day 15. However, enhanced numbers as well as enhanced frequencies of KLRG1^+^ CD8^+^ T cells were still observed on day 35 post infection although these differences were not significant. Cell numbers as well as the percentage of KLRG1^+^ CD8^+^ T cells were approximately doubled at this late point in time (day 35: 2.43x10^5^±3.70x10^4^, 2.27±0.27%; naïve: 1.16x10^5^±2.77x10^4^, 1.11±0.18%; Fig 1A, middle and right). In line with these findings a significantly enhanced proportion of CD8^+^ T cells expressed IFNγ as well as Granzyme B on day 7 post infection. These populations declined until day 15. The frequency of IFNγ producers, however, did not reach basal levels again (Fig 1B). Similar was also true for CD4^+^ T cells that were analyzed in parallel. A peak of enhanced frequencies of IFNγ-producing CD4^+^ T cells was detectable on day 7 post infection. The population of IFNγ-expressing CD4^+^ T cells declined until day 15 but did not return to basal level until day 35 (Fig 1C, left). These data show that C57BL/6 mice mount an efficient cytotoxic CD8^+^ T cell response and a CD4^+^ T_H_1 response that is characterized by the expression of IFNγ.

### CD8^+^ as well as CD4^+^ T cells are protective against *R*. *typhi*

We next asked whether CD8^+^ and/or CD4^+^ T cells exert protective functions *in vivo*. In a first approach we used C57BL/6 MHCII^-/-^ and C57BL/6 MHCI^-/-^ mice that either lack CD4^+^ T cells or CD8^+^ T cells. All of these mice were protected against *R*. *typhi*-induced disease and survived the infection more than 150 days (S1A Fig), demonstrating that CD8^+^ as well as CD4^+^ T cells are sufficient for protection.

**Fig 2:**
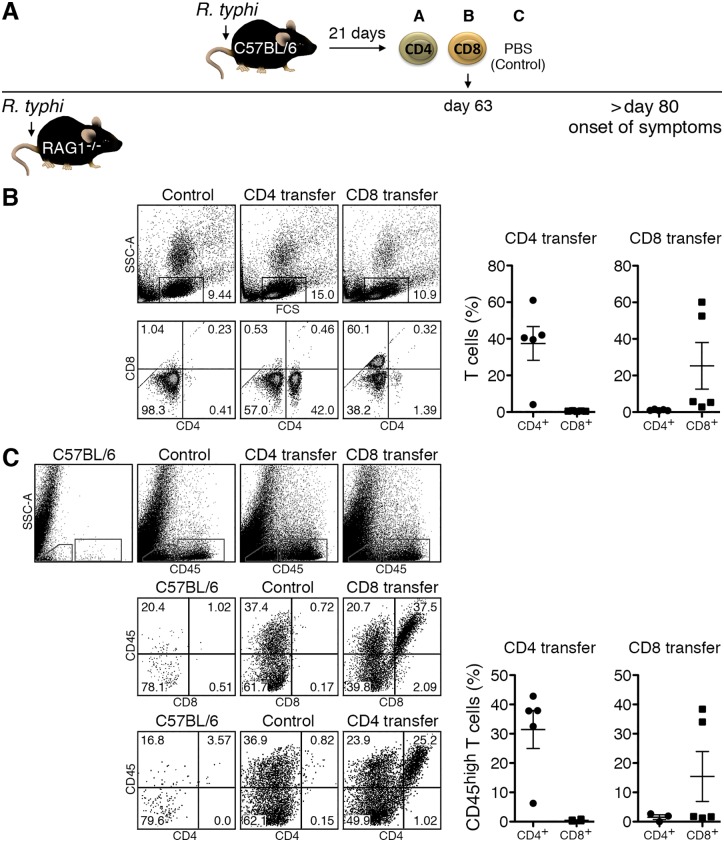
T cells enter the brain after adoptive transfer into *R*. *typhi*-infected C57BL/6 RAG1^-/-^ mice. CD4^+^ and CD8^+^ T cells were purified from C57BL/6 mice 21 days post infection to obtain immune T cells. T cells were adoptively transferred into C57BL/6 RAG1^-/-^ on day 63 post infection with *R*. *typhi*, which is approximately 20 days prior to the onset of disease. Control mice received PBS instead of T cells (**A**). Blood cells were analyzed for the presence of CD4^+^ and CD8^+^ T cells by flow cytometry on day 7 post T cell transfer by flow cytometry. Dot plots show representative stainings of the blood from a control mouse, a CD4^+^ and a CD8^+^ T cell recipient. Graphs show the percentage (y-axis) of CD4^+^ and CD8^+^ T cells as indicated on the x-axis among blood leukocytes in the groups of CD4^+^ and CD8^+^ recipient mice (**B**). Brain cells were analyzed for the presence of CD4^+^ and CD8^+^ T cells by flow cytometry gated on CD45^high^ cells. Representative stainings of a naïve C57BL/6 mouse that was used as an additional control, a *R*. *typhi*-infected C57BL/6 RAG1^-/-^ control mouse and a CD4^+^ and CD8^+^ T cell recipient are shown. Graphs show the percentage (y-axis) of CD4^+^ and CD8^+^ T cells as indicated on the x-axis among CD45^high^ cells in the brains of the groups of CD4^+^ and CD8^+^ recipient mice (**C**). Each symbol represents a single mouse.

Second, we performed adoptive T cell transfer into *R*. *typhi*-infected C57BL/6 RAG1^-/-^ mice to show whether CD8^+^ and CD4^+^ T cells would still be protective in an established *R*. *typhi*-infection. Because these animals are capable to control the bacteria for a long period of time, antigen availability may be low and not sufficient to elicit efficient T cell responses in time. Therefore, we decided to transfer isolated immune CD8^+^ and CD4^+^ T cells from *R*. *typhi*-infected C57BL/6 wild-type mice instead of T cells from naïve animals as these get activated much faster upon antigen recognition. CD8^+^ and CD4^+^ T immune cells were obtained from C57BL/6 wild-type mice on day 21 post *R*. *typhi* infection and first transferred into C57BL/6 RAG1^-/-^ mice either on day 45 or on day 55 post infection. None of these animals developed disease whether receiving CD8^+^ or CD4^+^ T cells and survived the infection. S1B Fig shows the results for the transfer experiment performed on day 55. We next adoptively transferred isolated immune CD4^+^ and CD8^+^ T cells into C57BL/6 RAG1^-/-^ mice later in infection on day 63, which is approximately 20–40 days prior to the usual onset of neurological symptoms. At this point in time the bacteria become detectable in the brain by qPCR. Fig 2A shows a schematic overview of the procedure. Control animals received PBS instead of T cells. Additional control groups of mice received either CD4^+^ or CD8^+^ T cells but were non-infected.

First, we analyzed if the transferred T cells were detectable in the blood of recipient mice and if the cells would enter the brain. These analyses were performed on day 7 post T cell transfer. In the T cell recipient C57BL/6 RAG1^-/-^ mice CD4^+^ as well as CD8^+^ T cells were clearly detectable in the blood of all animals that received the respective T cell population (Fig 2B). In the brain infiltrating cells can generally be distinguished from resident immune cells (mainly microglia) by the expression of high levels of CD45. CD45^high^ cells including T cells are virtually absent in the brain of naïve C57BL/6 wild-type mice (Fig 2C) and were not detectable in the brain of non-infected C57BL/6 RAG1^-/-^ mice that received either immune CD4^+^ or CD8^+^ T cells. In contrast, all *R*. *typhi*-infected C57BL/6 RAG1^-/-^ mice showed high numbers of CD45^high^ cellular infiltrates in the brain. Among these, CD4^+^ T cells were detectable at high frequencies in all CD4^+^ recipient mice (31.46±6.50%) while CD8^+^ T cells were present in the brain of only 2 out of 5 recipient mice at this point in time (36.20±2.20%) (Fig 2C) although all animals of this group showed low frequencies of CD8^+^ T cells in the blood (Fig 2B). These data demonstrate that immune T cells enter the CNS of *R*. *typhi*-infected C57BL/6 RAG1^-/-^ mice.

**Fig 3:**
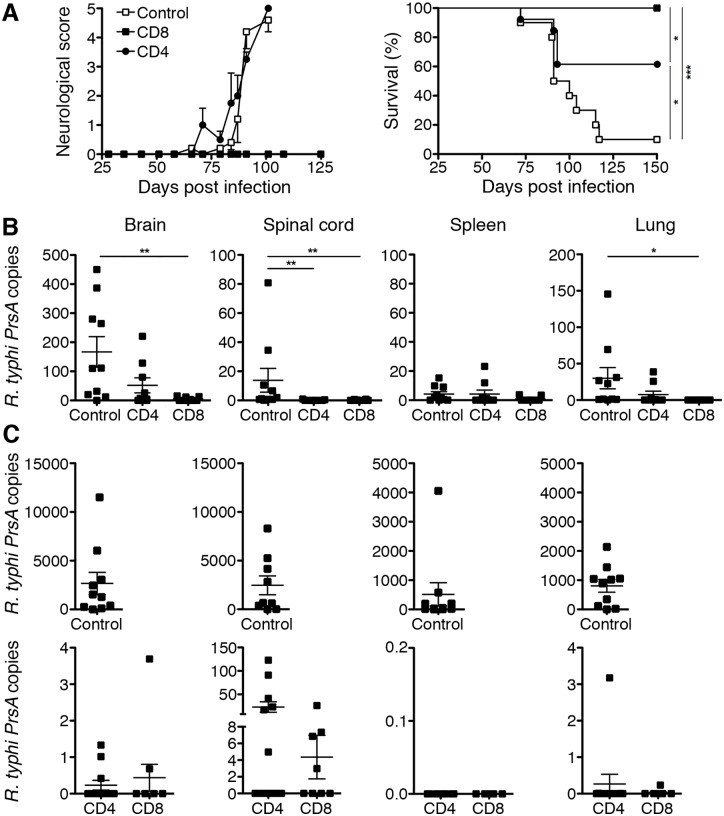
CD4^+^ T cells are protective but less efficient in bacterial elimination compared to CD8^+^ T cells. The health status of the mice was monitored by a neurological score at indicated points in time. The left graph shows the neurological score (y-axis) of *R*. *typhi*-infected C57BL/6 RAG1^-/-^ control animals (open squares; n = 5), CD4^+^ T cell recipients (black circles; n = 5) and CD8^+^ T cell recipients (black squares; n = 5) at indicated points in time (x-axis). The right graph shows the survival of each of these groups. Combined results from two independent experiments are shown (CD8^+^ recipients: n = 10; CD4^+^ T cell recipients: n = 13). Statistical analysis was performed with Log rank test. Asterisks indicate statistically significant differences between the indicated groups (*p<0.05, **p<0.01, ***p<0.001) (**A**). The bacterial load in the brain, spinal cord, spleen and lung (y-axis) of *R*. *typhi*-infected control animals, CD4^+^ and CD8^+^ T cell recipients (x-axis) was determined by qPCR on day 7 post transfer (**B**). The bacterial load was determined in the same tissues of *R*. *typhi*-infected control animals at the time of death (upper panel) and of CD8^+^ and CD4^+^ T cell recipients when the experiments were terminated (day 210 post infection; lower panel). In case of the CD4^+^ T cell recipients data from all animals including the five mice that succumbed to the infection are shown. These animals were negative for *R*. *typhi* (**C**). Each symbol represents a single mouse. Statistical analysis was performed with Kruskal-Wallis test and Dunn´s post test. Asterisks indicate statistically significant differences (*p<0.05, **p<0.01, ***p<0.001).

90% of the control C57BL/6 RAG1^-/-^ mice that were infected with *R*. *typhi* but received PBS instead of T cells succumbed to the infection between day 70 and 120, showing neurological symptoms such as tremor, ataxia and paralysis followed by body weight loss. These were evaluated by a neurological score (Fig 3A). Despite the late transfer on day 63, C57BL/6 RAG1^-/-^ mice that obtained CD8^+^ immune T cells did not show any signs of disease at any point in time. Moreover, all of these animals survived the infection. In contrast, approximately 40% of the C57BL/6 RAG1^-/-^ mice that obtained CD4^+^ immune T cells developed a neurological score and the same symptoms as *R*. *typhi*-infected C57BL/6 RAG1^-/-^ control mice including tremor and/or ataxia. Furthermore, disease progressed in these animals with similar kinetics as in control mice. The affected CD4^+^ T cell recipients lost weight and died within the same time frame as control mice (Fig 3A). The remaining CD4^+^ T cell recipients did not show symptoms of disease at any point in time and survived (Fig 3A).

These data demonstrate that CD4^+^ T cells are less protective against *R*. *typhi*-induced disease than CD8^+^ T cells when applied late in advanced infection.

### CD4^+^ T cells are less efficient in bacterial elimination than CD8^+^ T cells

Next, we performed *R*. *typhi*-specific qPCR from DNA of different organs from *R*. *typhi*-infected CD4^+^ and CD8^+^ T cell recipients and control mice as well as from non-infected CD4^+^ and CD8^+^ T cell recipients to quantify the bacterial load. Analysis was performed for all groups on day 7 post T cell transfer or treatment with PBS, respectively. Furthermore, bacterial load was determined at the time of death in control animals and CD4^+^ T cell recipients that did not survive the infection. Organs from CD8^+^ and surviving CD4^+^ T cell recipients were taken on day 210 when the experiment was terminated. As described previously, the bacteria were predominantly found in the brain of *R*. *typhi*-infected C57BL/6 RAG1^-/-^ mice but were also present at lower amounts in the spinal cord, spleen and lung (Fig 3B and 3C upper panel) and virtually absent in the liver. The average copy numbers actually measured in each organ of each group are shown in Table 1.

**Table 1:** Bacterial content in tissues from *R*. *typhi*-infected C57BL/6 RAG1^-/-^ mice.

	**Day 7 post transfer**		
	**Control**	**CD4**^**+**^ **transfer**	**CD8**^**+**^ **transfer**
**Brain**	166.60 ± 52.57	51.80 ± 25.84	4.88 ± 2.21
**Spinal cord**	13.82 ± 8.15	0.17 ± 0.11	0.10 ± 0.11
**Spleen**	4.23 ± 1.68	4.23 ± 2.70	0.82 ± 0.53
**Lung**	29.96 ± 14.61	7.52 ± 4.81	0.03 ± 0.01
**Liver**	0.11 ± 0.05	0.08 ± 0.06	0.08 ± 0.05
	**Time of death and end of experiment**		
	**Control**	**CD4**^**+**^ transfer*	**CD8**^**+**^ transfer**
**Brain**	2960.00 ± 1235.00	0.23 ± 0.13	0.44 ± 0.37
**Spinal cord**	2467.00 ± 970.40	23.15 ± 11.04	4.35 ± 2.61
**Spleen**	515.4 ± 397.3	0.00	0.00
**Lung**	808.30 ± 218.7	0.26 ± 0.26	0.02 ± 0.02
**Liver**	40.35 ± 24.36	0.28 ± 0.28	0.00

*Tissues from all CD4^+^ T cell recipients including those that died between day 70 and day 162 (4 animals) were analyzed. Tissues from surviving animals (7 mice) were taken when the experiment was terminated on day 210 after *R*. *typhi* infection.

**All CD8^+^ T cell recipients survived the infection. Tissues from these animals were taken when the experiment was terminated on day 210 after *R*. *typhi* infection.

The table shows the *R*. *typhi PrsA* gene copy numbers measured in 20 ng tissue DNA (mean± SEM) from the animals shown in Fig 3B and 3C.

In mice that had received CD8^+^ immune T cells the bacteria were already significantly reduced and almost eliminated in all analyzed tissues as early as at day 7 post T cell transfer (Fig 3B and Table 1). Furthermore, on day 7 post transfer mice that had received CD4^+^ T cells also showed reduced bacterial loads in the brain, spinal cord and lung but not in the spleen where the bacterial content was generally low (Fig 3B).

On day 210 when the experiment was terminated *R*. *typhi* was not detectable anymore in six out of ten CD8^+^ T cell recipient mice. However, the remaining four animals showed low amounts of bacteria predominantly in the spinal cord. Three of these mice also had few bacteria in the brain while *R*. *typhi* was not detectable in the spleen and lung (Fig 3C lower panel and Table 1). Surprisingly, the bacteria were not present anymore in the five CD4^+^ recipients that succumbed to the infection. However, six mice of the seven surviving animals of this group had bacteria in the spinal cord at the end of the experiment on day 210. Three of these animals also showed low copy numbers in the brain and one mouse in the lung (Fig 3C lower panel and Table 1). The bacteria were generally not detectable in the organs from non-infected animals that received either immune CD4^+^ or CD8^+^ T cells (S2 Fig), excluding that a possible co-transfer of low amounts of contaminating *R*. *typhi* might have contributed to infection.

These data clearly demonstrate that immune CD4^+^ T cells are capable to eradicate *R*. *typhi* although bacterial elimination by CD8^+^ T cells is much more efficient and faster. They further show that both CD8^+^ and CD4^+^ T cells are capable to control persisting *R*. *typhi* and prevent recurrence of disease.

### CD4^+^ T cells support bactericidal function and inflammatory responses of MΦ and microglia in the CNS of *R*. *typhi*-infected C57BL/6 RAG1^-/-^ mice

**Fig 4:**
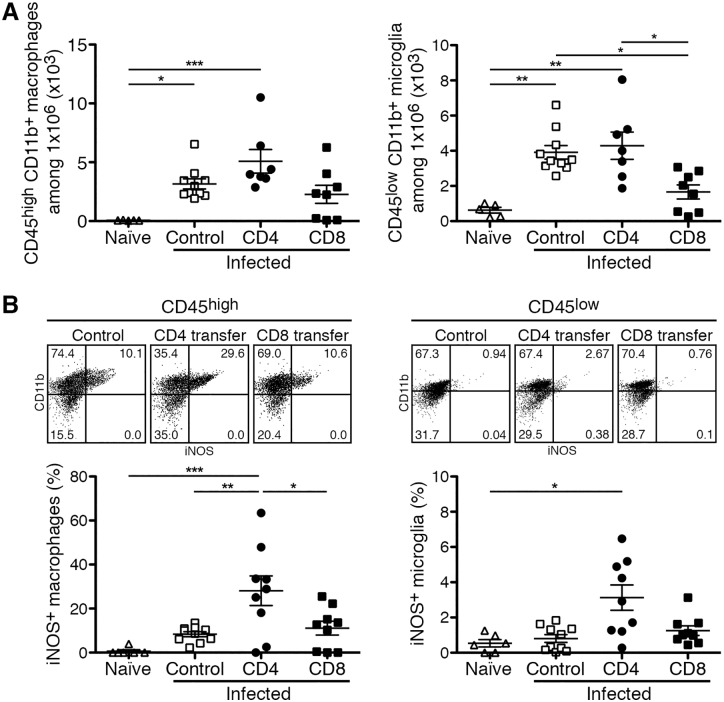
CD4^+^ T cells induce iNOS expression in brain infiltrating MΦ as well as in resident microglia (day 7 post transfer). Numbers of CD45^high^CD11b^+^ infiltrating MΦ (y-axis, left) and CD45^low^CD11b^+^ microglia (y-axis, right) were determined in the brains of naïve C57BL/6 RAG1^-/-^, *R*. *typhi*-infected control mice and *R*. *typhi*-infected CD4^+^ and CD8^+^ T cell recipients as indicated on the x-axis. Analysis was performed by flow cytometry. 1×10^6^ events were analyzed (**A**). Brain cells were further analyzed for intracellular iNOS expression by flow cytometry. A representative dot plot is shown for the staining of iNOS and CD11b for CD45^high^ infiltrating cells (left) and CD45^low^ resident cells (right). Graphs show the calculated percentage of iNOS^+^ cells among CD45^high^CD11b^+^ infiltrating MΦ (left) and resident CD45^low^CD11b^+^ microglia (right) (y-axis) for each group (x-axis) (B). Each dot represents a single mouse. Statistical analysis was performed with Kruskal-Wallis test and Dunn´s post test. Asterisks indicate statistically significant differences (*p<0.05, **p<0.01, ***p<0.001).

We have previously shown that *R*. *typhi*-induced CNS inflammation is characterized by the expansion of microglia as well as by the infiltration of MΦ from the periphery and that the latter harbor the bacteria [48]. Therefore, we further performed flow cytometric analyses of the brain to quantify microglia and MΦ. As observed previously a significant increase in the numbers of microglia (CD45^low^CD11b^+^) as well as of infiltrating MΦ (CD45^high^CD11b^+^) was detectable in the brain of *R*. *typhi*-infected control C57BL/6 RAG1^-/-^ mice (microglia: 3913±386.7; MΦ: 3160±433.3 among 1×10^6^ events) compared to naïve mice (microglia: 625.6±162.8) where infiltrating MΦ were virtually absent (MΦ: 68.19±10.42) as expected. Counts of microglia were not signicantly altered in CD4^+^ T cell recipients (4285±774.4) while an enhanced infiltration of MΦ, although not significant, was observed (5082±995.3) compared to infected control mice. In contrast, mice that had received CD8^+^ T cells showed significantly reduced numbers of microglia (1663±397.4) and numbers of infiltrating MΦ were unchanged compared to infected control mice (2272±767.4) (Fig 4A).

We further analyzed the activation status of microglia and infiltrating MΦ. For this purpose we performed intracellular staining of inducible nitric oxide synthase (iNOS). This enzyme is usually expressed by activated MΦ and important for the killing of intracellular bacteria by catalyzing the generation of nitric oxide (NO) [70]. We previously described that a significantly enhanced proportion of infiltrating MΦ but not microglia express iNOS in *R*. *typhi*-infected C57BL/6 RAG1^-/-^ mice at the time of death [48]. Here, analyses were performed much earlier 7 days post T cell transfer (day 70 post infection) which is before the usual onset of symptoms. Although enhanced frequencies of iNOS-expressing infiltrating MΦ were observed in the brain of C57BL/6 RAG1^-/-^ control mice (8.375±1.118%) compared to naïve mice (0.6533±0.6533%), these differences were not significant at this point in time (Fig 4B). The frequency of iNOS-expressing infiltrating MΦ was not significantly altered in mice that had received CD8^+^ T cells (11.1±3.156%). In contrast, the transfer of CD4^+^ T cells led to a significantly increased proportion of iNOS-expressing MΦ (28.10±6.713%). Furthermore, the presence of CD4^+^ T cells led to a significantly increased frequency of iNOS-expressing microglia (3.132±0.717%). These cells normally do not express iNOS in *R*. *typhi*-infection of C57BL/6 RAG1^-/-^ mice [48]. In line with these previous findings, iNOS-expressing microglia were not detectable in *R*. *typhi*-infected control mice (0.8054±0.2214%) compared to 0.5350±0.2074% in naïve mice. Furthermore, enhanced frequencies of iNOS-expressing microglia were not present in mice that had received CD8^+^ T cells (1.254±0.2734%) (Fig 4B).

**Fig 5:**
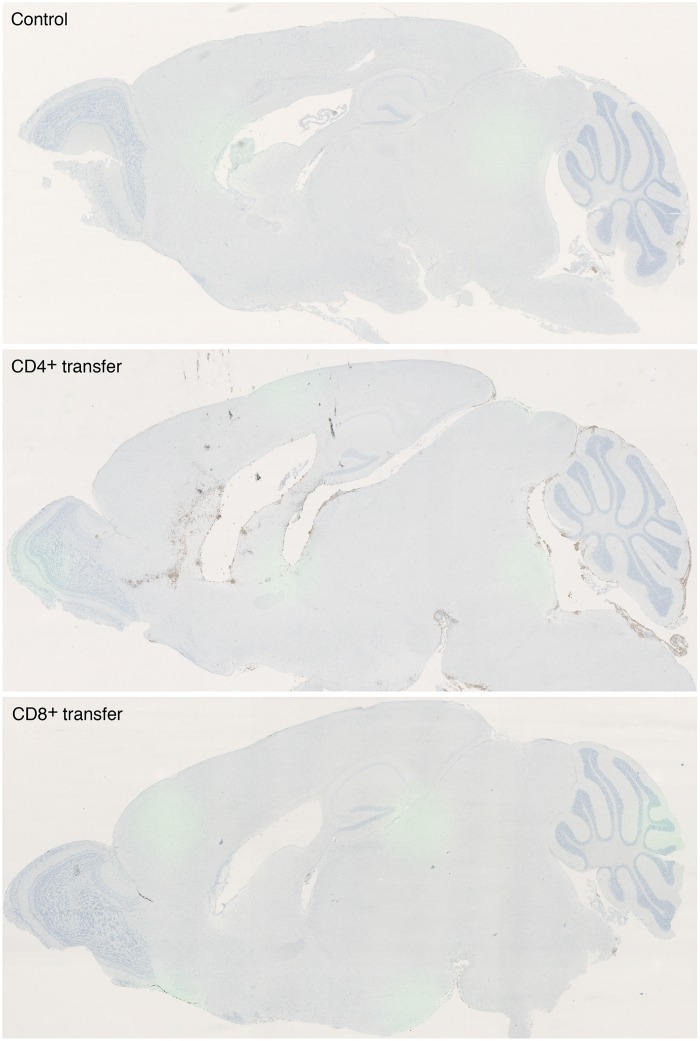
Representative overviews of the detection of iNOS-expressing cells in the brain of *R*. *typhi*-infected C57BL/6 RAG1^-/-^ control mice, CD4^+^ and CD8^+^ recipients (day 7 post transfer). Sagittal sections of the brains from *R*. *typhi*-infected C57BL/6 RAG1^-/-^ control mice, CD4^+^ and CD8^+^ recipients were stained for iNOS. Images were taken at 2fold magnification. At this point in time, numbers of iNOS-expressing cells in the brain of control animals was still low. Similar was observed in CD8^+^ T cell recipients while CD4^+^ T cell recipients showed strongly increased numbers of iNOS expressing cells. These were found predominantly at the ventricle borders and in the pia mater of the cerebellum.

**Fig 6:**
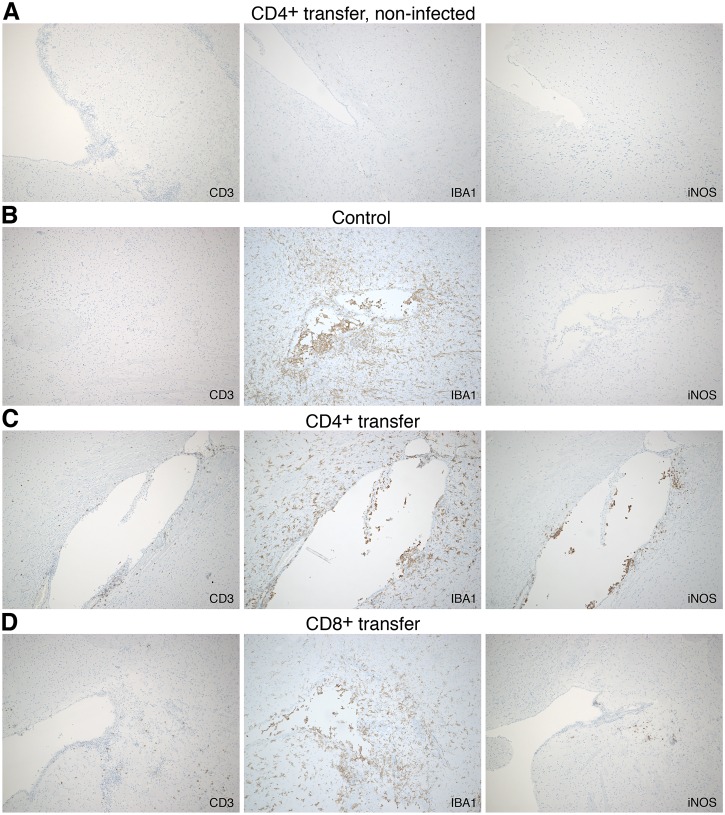
Detection of infiltrating T cells, IBA1^+^ cells and iNOS expression in sagittal sections of the brains from *R*. *typhi*-infected C57BL/6 RAG1^-/-^ control mice, CD4^+^ and CD8^+^ recipients (day 7 post transfer). Sagittal sections were further stained for CD3 to detect infiltrating T cells, for IBA1 that is expressed on infiltrating MΦ as well as by microglia and for iNOS. Sections from the brains of CD4^+^ T cell recipients that were not infected were used as additional control (**A**). Representative stainings of the brain from a *R*. *typhi*-infected C57BL/6 RAG1^-/-^ control mouse (**B**), a *R*. *typhi*-infected mouse that received CD4^+^ T cells (**C**) and a CD8^+^ T cell recipient (**D**) are shown.

Enhanced numbers of iNOS-expressing cells were also detectable in the CNS of CD4^+^ T cell recipients in histological stainings. Fig 5 shows representative overview stainings of iNOS in the brain from a *R*. *typhi*-infected control mouse, a CD4^+^ and a CD8^+^ T cell recipient 7 days post transfer. iNOS-expressing cells were hardly detectable in control mice and animals that received CD8^+^ T cells. In CD4^+^ T cell recipients locally accumulating iNOS-expressing cells were found at the ventricle borders as well as in the parenchyma and the pia mater of the cerebellum (Fig 5). Furthermore, infiltrating CD4^+^ and CD8^+^ T cells were observed in *R*. *typhi*-infected recipients mainly at the ventricle borders but also present in the parenchyma (Fig 6C and 6D) while T cells were not detectable in the brain of non-infected mice that received CD8^+^ or CD4^+^ T cells (Fig 6A). Infiltrating T cells were accompanied by accumulating IBA1^+^ cells, most likely infiltrating MΦ. Only the brains of mice that had received CD4^+^ T cells showed high numbers of iNOS-expressing IBA1^+^ cells that colocalized with infiltrating T cells (Fig 6C).

**Fig 7:**
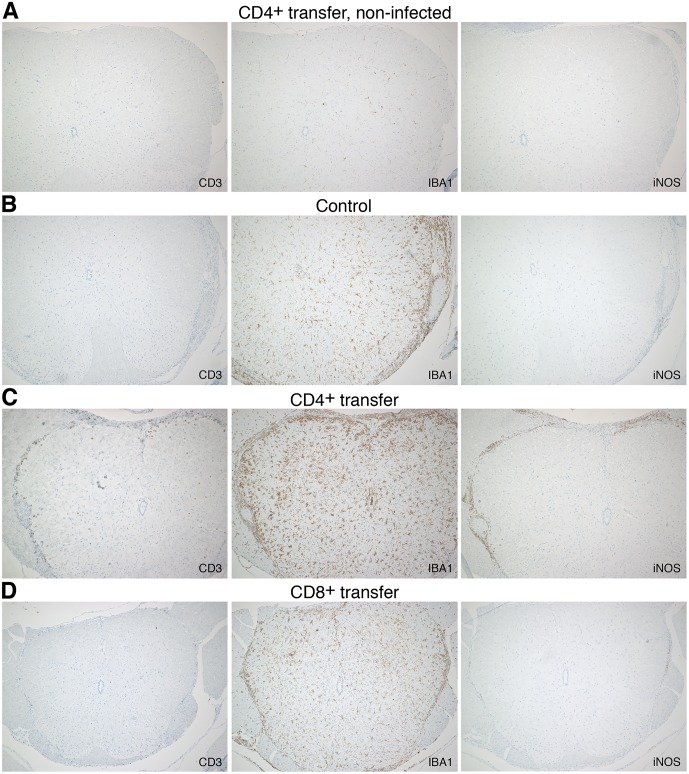
Detection of infiltrating T cells, IBA1^+^ cells and iNOS expression in sections of the spinal cord from *R*. *typhi*-infected C57BL/6 RAG1^-/-^ control mice, CD4^+^ and CD8^+^ recipients (day 7 post transfer). Lumbar spinal cord sections of *R*. *typhi*-infected C57BL/6 RAG1^-/-^ mice that received CD4^+^ or CD8^+^ T cells and from *R*. *typhi*-infected control animals were stained for the presence of T cells (CD3), MΦ and microglia (IBA1) and iNOS. Sections from the spinal cord of non-infected CD4^+^ T cell recipients were used as additional control (**A**). Representative stainings of the spinal cord from a *R*. *typhi*-infected C57BL/6 RAG1^-/-^ control mouse (**B**), a *R*. *typhi*-infected mouse that received CD4^+^ T cells (**C**) and a CD8^+^ T cell recipient (**D**) are shown.

Similar observations were made in histological stainings of the spinal cord 7 days post transfer. In *R*. *typhi*-infected control mice that had received PBS instead of T cells, accumulating IBA1^+^ cells were observed in the gray and white matter (Fig 7B). These cells most likely represent microglia. In addition, accumulating IBA1^+^ cells, most probably infiltrating MΦ, were detectable in peripheral areas, presumably the pia mater and subarachnoid space where the bacteria reside [48]. CD4^+^ and CD8^+^ T cell infiltrates were also predominantly detectable in these areas while only few T cells were observed in the gray and white matter in recipient mice (Fig 7C and 7D). In *R*. *typhi*-infected control mice iNOS-expressing cells were still rare at this point in time of infection (Fig 7B) and also hardly detectable in mice that received CD8^+^ T cells (Fig 7D). In contrast, CD4^+^ T cell infiltrates were associated with iNOS-expressing infiltrating IBA1^+^ MΦ. Exclusively in the spinal cord of animals that had received CD4^+^ T cells few iNOS-expressing cells were also detectable in the gray and white matter (Fig 7C). Neither T cell infiltration nor accumulation of IBA1^+^ cells and iNOS expression occurred in the spinal cord of non-infected mice that had received CD8^+^ or CD4^+^ T cells (Fig 7A).

Collectively, these results demonstrate that CD4^+^ T cells significantly induce iNOS-expression not only in infiltrating MΦ but also in microglia, indicating enhanced bactericidal function of these cells and enhanced inflammatory response in the CNS of these animals.

### *R*. *typhi* does not activate MΦ in a classical manner

**Fig 8:**
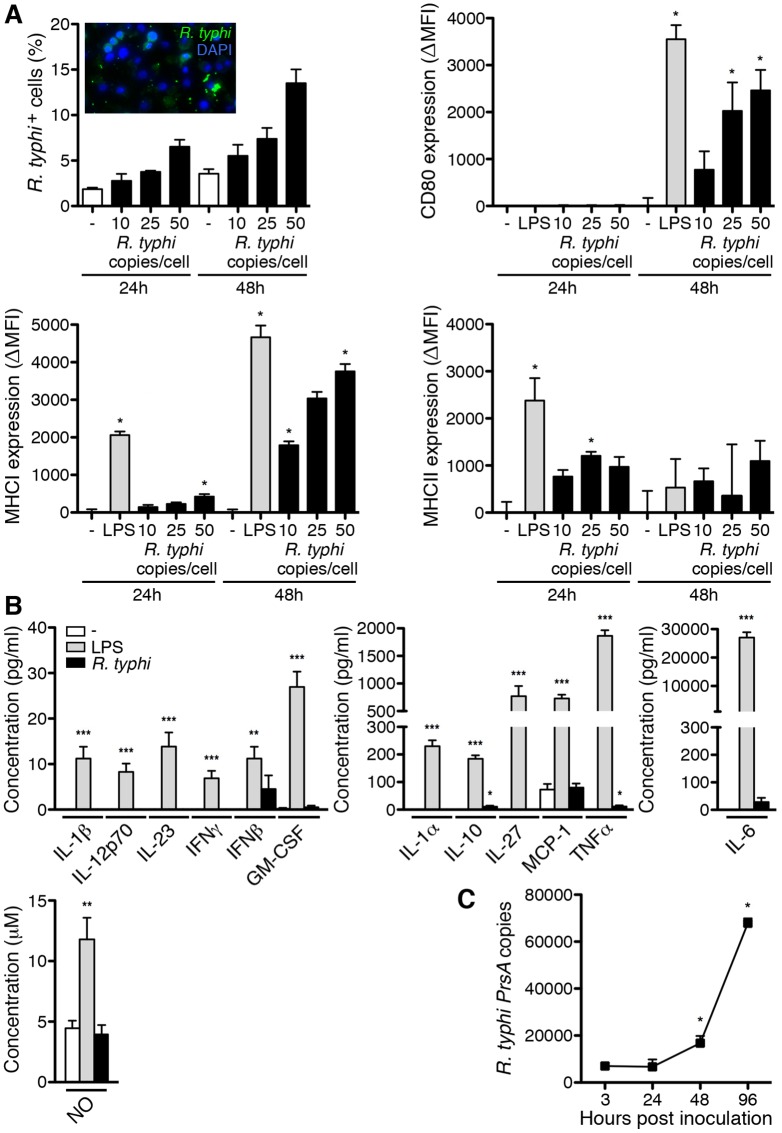
MΦ do not react to *R*. *typhi* in a classical manner and are incapable to kill the bacteria *in vitro*. bmMΦ were left untreated, stimulated with LPS (500 ng/ml) as a control or infected with indicated amounts of *R*. *typhi* copies, stimulated with LPS (500 ng/ml) or left untreated (-) as indicated on the x-axis. Bacterial content, the expression of CD80, MHCI and MHCII was analyzed after 24h and 48h by flow cytometry. In addition, *R*. *typhi* was stained for immunofluorescence microscopy 48h after infection (insertion). Graphs show the percentage of *R*. *typhi*-positive cells (y-axis) and the mean fluorescence intensity (MFI) minus the mean MFI of untreated cells (ΔMFI, y-axis). The graphs show combined results from the stimulation of bmMΦ derived from four individual C57BL/6 mice. Statistical analysis was performed with Kruskal-Wallis test and Dunn´s post test. Asterisks indicate statistically significant differences compared to untreated cells (*p<0.05) (**A**). The concentration (y-axis) of the indicated cytokines and NO (x-axis) was quantified in the supernatants from untreated (white bars) or LPS-stimulated bmMΦ (gray bars) and from bmMΦ cultures that were infected with 50 *R*. *typhi* copies per cell (black bars). Cytokines and NO were determined at 48h post inoculation. Statistical analysis was performed with Kruskal-Wallis test and Dunn´s post test. Asterisks indicate statistically significant differences compared to untreated cells (*p<0.05**, *p*<0.01, ***p<0.001) (**B**). bmMΦ were infected with 5 *R*. *typhi* copies per cell. Medium was exchanged after 3h and cells were further incubated for 96h. Copy numbers of *R*. *typhi* (y-axis) were determined at indicated points in time (x-axis) by qPCR. The graph shows combined results from the stimulation of bmMΦ from four individual C57BL/6 mice. Statistical analysis was performed with Mann-Whitney U test. Asterisks indicate statistically significant differences compared to non-infected control cells (*p<0.05) (**C**).

MΦ represent target cells for *R*. *typhi* that infiltrate the CNS of infected C57BL/6 RAG1^-/-^ mice [48]. Because these cells were found to express iNOS *in vivo*, we further asked if *R*. *typhi* would infect MΦ *in vitro* and how these cells would react to the bacteria. To this end we incubated bmMΦ with titrated amounts of *R*. *typhi* or stimulated the cells with LPS while control cells were left untreated. First, we assessed bacterial uptake. After 48h of incubation approximately 15% of the bmMΦ that were infected with 50 bacterial particles per cell were positive for *R*. *typhi* as detected by flow cytometry (Fig 8A). In fact, at least one bacterium was detectable in the cytosol of every cell 48h after infection in immunofluorescent stainings (Fig 8A, insertion) demonstrating that all cells had been in contact with *R*. *typhi*. We further analyzed the expression of MHCI, MHCII and costimulatory molecules on the cell surface 24h and 48h after inoculation. LPS-stimulated and untreated MΦ were used as a control. LPS stimulation led to a significant up-regulation of CD80 and MHCI with maximum expression at 48h while MHCII expression was temporarily enhanced at 24h (Fig 8A). *R*. *typhi*-infected MΦ up-regulated the expression of CD80, MHCI and MHCII in a dose-dependent manner with similar kinetics (Fig 8A) demonstrating MΦ activation. We further assessed cytokine and NO production in cultures that were infected with the highest dose of *R*. *typhi* (50 bacterial copies per cell). As expected, LPS significantly induced the release of several cytokines as well as the production of NO (Fig 8B). Surprisingly, *R*. *typhi*-infected bmMΦ hardly released any cytokines. Only very low amounts of IL-6 (28.4±15.4 pg/ml), TNFα (11.1±4.6 pg/ml) and IL-10 (10.9±3.5 pg/ml) were detectable. These were negligible compared to LPS-induced levels of these cytokines (IL-6: 27039±1896 pg/ml, TNFα: 1865±101,7 pg/ml, IL-10: 184.6±12.5 pg/ml). Furthermore, the release of bactericidal NO was not induced in *R*. *typhi*-infected bmMΦ cultures (Fig 8B). These data show that MΦ do not react to *R*. *typhi* in a classical manner. The observation that *R*. *typhi* does not induce the production of bactericidal NO further led to the question whether bmMΦ are capable to kill *R*. *typhi in vitro*. Therefore, we analyzed bacterial growth in bmMΦ cultures. Bacterial content was quantified by qPCR at indicated points in time. Significantly enhanced copy numbers were detected already at 48h and bacteria further increased until 96h post inoculation (Fig 8C), demonstrating that MΦ are incapable to eliminate the bacteria *in vitro* and that *R*. *typhi* grows within these cells.

### CD4^+^ T cells promote bacterial killing by MΦ

**Fig 9:**
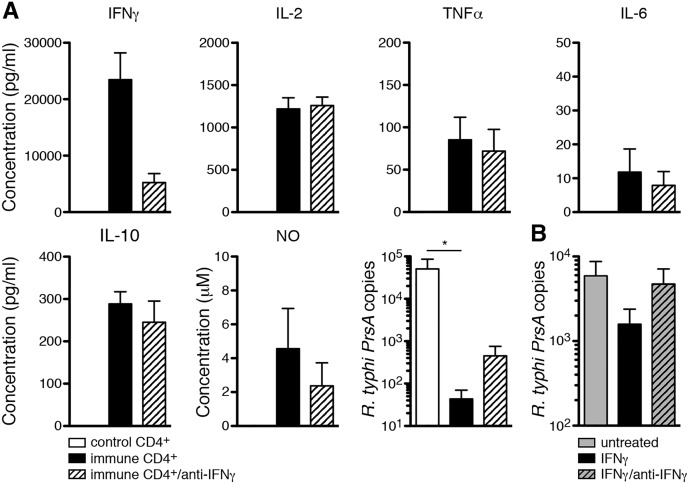
Immune CD4^+^ T cells and IFNγ activate bmMΦ for bacterial killing *in vitro*. 2×10^6^ bmMΦ were infected with 5 copies of *R*. *typhi* per cell. Medium was exchanged 3h post infection. 24h after infection 1.5×10^6^ purified CD4^+^ T cells from either PBS-treated control (white bars) or *R*. *typhi*-infected C57BL/6 wild-type mice (day 7 post infection; black bars) were added. IFNγ was neutralized by simultaneous addition of anti- IFNγ (1 μg/ml; striped bar). Cytokines and NO were quantified in the cell culture supernatant (y-axis) 96h post bmMΦ infection (48h after addition of T cells or IFNγ) and bacterial growth was assessed by qPCR 96h post bmMΦ infection. Graph shows combined results from two independent experiments for each of which T cells from 6 individual control or *R*. *typhi*-infected mice were used (**A**). 2×10^6^ bmMΦ were infected with 5 copies of *R*. *typhi* per cell. Medium was exchanged 3h post infection and either replaced by unconditioned medium (gray bar), medium containing recombinant IFNγ (10 U/ml; black bar) or the same amount of recombinant IFNγ that was pre-incubated with neutralizing anti-IFNγ (1 μg/ml; gray striped bar). Bars show combined results from two independent experiments for each of which MΦ from 3 individual C57BL/6 mice were used (**B**). Statistical analysis was performed with Kruskal-Wallis test followed by Dunn´s post test. Asterisks indicate statistically significant differences (*p<0.05, ***p<0.001).

Having shown that CD4^+^ T cells produce IFNγ in *R*. *typhi* infection and enhance MΦ activation *in vivo*, we finally asked whether immune CD4^+^ T cells or IFNγ can activate bmMΦ for bacterial elimination *in vitro*. To this end, bmMΦ were infected with *R*. *typhi in vitro* as described in the previous section. 24h after inoculation either immune CD4^+^ T cells or CD4^+^ T cells from non-infected C57BL/6 control animals were added. In addition, IFNγ was neutralized by anti-IFNγ. The release of cytokines and NO and bacterial growth was assessed 96h post bmMΦ infection. Control CD4^+^ T cells did not react to infected bmMΦ with the release of detectable amounts of cytokines and did not induce the release of NO whereas cultures containing immune CD4^+^ T cells produced very high amounts of IFNγ (23456±4758 pg/ml) and IL-2 (1219.7±130.8 pg/ml). In addition, low levels of TNFα (85.2±26.7 pg/ml), IL-6 (11.8±6.8 pg/ml), IL-10 (288.6±28.4 pg/ml) and bactericidal NO (3.9±2 μM) were detectable in cultures containing immune CD4^+^ T cells (Fig 9A). Despite the high levels of IFNγ the cytokine could be neutralized to a certain extent (5203±1606 pg/ml). Neutralization of IFNγ, however, had no significant effect on the production of the other cytokines or NO (Fig 9A). Furthermore, immune CD4^+^ T cells nearly completely inhibited bacterial growth (43±26 *R*. *typhi PrsA* copies) compared to cultures with control CD4^+^ T cells (17320±13458 *R*. *typhi PrsA* copies) (Fig 9B). This effect was in part, although not significantly, inhibited by the addition of neutralizing anti-IFNγ antibody (451±305 copies) (Fig 9A). To further elucidate the role of IFNγ, we incubated infected and non-infected bmMΦ with recombinant IFNγ. The cytokine alone did not induce the release of detectable amounts of cytokines or NO in bmMΦ, whether infected or not. However, IFNγ partially inhibited bacterial growth (2685±317 copies) compared to untreated infected bmMΦ cultures (7457±1261 copies). This effect was abolished by pre-incubation of IFNγ with neutralizing anti-IFNγ antibody (8200±1190 copies), demonstrating that it relies on the biological activity of the cytokine (Fig 9B). Collectively, these results demonstrate that immune CD4^+^ T cells enhance bacterial elimination by activating bactericidal functions of MΦ which is at least in part mediated by IFNγ.

## Discussion

We recently described that *R*. *typhi* shows a neurotropism in C57BL/6 RAG1^-/-^ mice that lack adaptive immunity. In these mice, *R*. *typhi* re-appears months after infection predominantly in the CNS and causes severe CNS inflammation and lethal paralysis. Employing this model and adoptive transfer we describe here that CD8^+^ and CD4^+^ T cells enter the CNS and that both T cell populations are protective against this infection and *R*. *typhi*-induced disease.

C57BL/6 wild-type mice mount a classical CD4^+^ T_H_1 T cell response that is characterized by IFNγ expression in response to *R*. *typhi* infection. In addition, functional cytotoxic CD8^+^ T cells expressing IFNγ and Granzyme B, an effector molecule that is crucial for target cell killing by rapid induction of apoptosis [71], were generated. Both IFNγ expression in CD4^+^ and CD8^+^ T cells as well as Granzyme B expression in CD8^+^ T cells peaked on day seven post infection which was consistent with the expression of CD11a and KLRG1 on CD8^+^ T cells, demonstrating that these cells were antigen-experienced effector cells. In line with these findings KLRG1 was expressed on CD8^+^ T cells with similar kinetics in C3H/HeN mice upon *R*. *typhi* infection [72]. Furthermore, enhanced cytotoxic CD8^+^ T cell responses were observed in *R*. *conorii*- and *R*. *australis*-infected C3H/HeN mice peaking at day 10 post infection [55]. T cell response to *R*. *typhi* in C57BL/6 mice declined until day 15 but did not reach basal levels again until day 35. This is consistent with the observation that *R*. *typhi* persists in these mice [48] and suggests that a certain level of activated T cells is needed for durable control of the bacteria.

Here we demonstrate that CD4^+^ T cells are sufficient for protection against *R*. *typhi* infection. Neither CD4^+^ T cell deficient C57BL/6 MHCII^-/-^ nor CD8^+^ T cell-deficient C57BL/6 MHCI^-/-^ mice developed disease and survived the infection. We further performed adoptive transfer of immune T cells isolated from C57BL/6 wild-type mice into *R*. *typhi*-infected C57BL/6 RAG1^-/-^. Transfers were performed at a point in time when the bacteria already start to dramatically increase in the brain, the organ with the highest bacterial load in these animals [48]. T cells for transfer were isolated from *R*. *typhi*-infected C57BL/6 wild-type mice on day 21 post infection. These mice are highly resistant to the infection with *R*. *typhi* and other rickettsiae. Pure T cells from these mice were used for transfer. Because T cells are not target cells for the bacteria it is highly unlikely that significant amounts of bacteria were co-transferred and might have established a superinfection. In line with that, the bacteria were not detectable in the organs of non-infected animals that received either immune CD4^+^ or CD8^+^ T cells. Moreover, both immune CD8^+^ and CD4^+^ T cells entered the brain in *R*. *typhi*-infected C57BL/ RAG1^-/-^ mice but not in non-infected animals, efficiently eliminated the bacteria and were protective. The survival rate of CD4^+^ T cell recipients, however, was lower than that of CD8^+^ T cell recipients, indicating that CD4^+^ T cells are less efficient in protecting animals with already advanced infection. Generally, CD8^+^ T cells were absent in CD4^+^ recipients and vice versa (Fig 2B and 2C) so that a contribution of the other party to the effects discussed in the following can be excluded.

C57BL/6 RAG1^-/-^ CD8^+^ T cell recipients eliminated *R*. *typhi* very quickly from the brain as well as from other organs in established *R*. *typhi*-infection in C57BL/6 RAG1^-/-^ mice. Already 7 days after the transfer of CD8^+^ T cells, *R*. *typhi* was not detectable anymore in CD8^+^ T cell recipients. Moreover, in some mice CD8^+^ T cells were not detectable anymore in the brain already on day 7 after transfer although the cells were still present in the blood. This may indicate bacterial elimination and an already declining immune response in these animals while bacterial load might have been higher in those mice that still showed CD8^+^ T cell infiltrates and, thus, ongoing immune response. In the end, adoptive transfer of CD8^+^ T cells completely prevented disease in all animals, demonstrating that CD8^+^ T cells alone are sufficient for protection. So far, a protective function of CD8^+^ T cells against *R*. *typhi* has been only demonstrated in the C3H/HeN infection model where the depletion of CD8^+^ T cells resulted in enhanced bacterial load and pathology [57] while the role of cytotoxic CD8^+^ T cells has been studied in more detail in mouse models of the infection with SFG *Rickettsiae*. C3H/HeN mice depleted of CD8^+^ T cells and challenged with a normally sublethal dose of *R*. *conorii* died or remained persistently infected. Moreover, adoptive transfer of immune CD8^+^ T cells protected C3H/HeN mice against a lethal challenge with *R*. *conorii* [56]. In addition, C57BL/6 MHCI^-/-^ mice that lack CD8^+^ T cells were highly susceptible to a lethal outcome of *R*. *australis* infection [55]. Defense against *R*. *australis* is largely mediated by the cytotoxic activity of CD8^+^ T cells rather than IFNγ as C57BL/6 Perforin^-/-^ mice showed enhanced susceptibility to this infection compared to wild-type mice. Furthermore, adoptive transfer of CD8^+^ T cells from C57BL/6 IFNγ^-/-^ mice into *R*. *australis*-infected C57BL/6 MHCI^-/-^ still reduced bacterial load [55]. These observations indicate a critical function of cytotoxic CD8^+^ T cells in defense against rickettsial infections and our results further show that CD8^+^ T cells alone can provide long-term control of persisting *R*. *typhi* and prevent recurrence of disease.

However, C57BL/6 MHCI^-/-^ mice that lack CD8^+^ T cells were not susceptible to *R*. *typhi* infection and did not develop symptomatic disease even until 150 days post infection. Moreover, adoptive transfer of CD4^+^ T cells from immune mice into C57BL/6 RAG1^-/-^ with advanced *R*. *typhi* infection still protected at least 60% of the mice from disease and death. In fact, the bacteria were efficiently eliminated from the CNS in those CD4^+^ T cell recipients that succumbed to the infection. Collectively, these results demonstrate for the first time that CD4^+^ T cells alone are sufficient to protect against *R*. *typhi*-induced disease as long as they are present in time. Furthermore, CD4^+^ T cells are as efficient as CD8^+^ T cells in providing long-term control of persisting *R*. *typhi* and are also capable to prevent recurrence of disease. So far, a protective function of CD4^+^ T cells has only been demonstrated in the infection of mice with SFG *Rickettsiae*. Here, adoptive transfer of immune CD4^+^ T lymphocytes protected C3H/HeN mice against challenge with a lethal dose of *R*. *conorii* [56]. C3H/HeN mice depleted of CD4^+^ T cells underwent a comparable course of disease in a sublethal infection with *R*. *conorii* as control mice. The mice cleared the infection and recovered [56]. This situation is similar to the infection of CD4^+^ T cell-deficient C57BL/6 MHCII^-/-^ mice with *R*. *typhi* where CD8^+^ T cells are present and can compensate for the absence of CD4^+^ T cells.

Surprisingly, although both adoptively transferred immune CD8^+^ and CD4^+^ T cells managed to almost completely eliminate the bacteria from the organs of *R*. *typhi*-infected C57BL/6 RAG1^-/-^ mice in the first days to weeks after transfer, the bacteria were again detectable by qPCR predominantly in the brain and spinal cord of some animals when the experiments were terminated. We interprete these findings as follows: Transferred T cells strongly react to *R*. *typhi* immediately after transfer into infected C57BL/6 RAG1^-/-^ mice which is reflected by the strong induction of iNOS in CD4^+^ T cell recipients. This immediate response is obviously strong enough to reduce the bacterial amounts below qPCR detection limit in the initial phase after transfer. Later in the chronic phase of infection (until day 210) the T cell response calms down. In this situation a certain threshold of either activated CD4^+^ or CD8^+^ T cells as observed in *R*. *typhi*-infected C57BL/6 mice (Fig 1) is capable to prevent fatal bacterial outgrowth and to keep bacterial amounts at a low level that is detectable in some animals by qPCR. The co-action of both cell populations, however, is obviously more efficient in bacterial suppression as numbers of persisting *R*. *typhi* in infected C57BL/6 wild-type mice are much lower and not detectable by qPCR [48].

The induction of IFNγ-producing CD4^+^ T_H_1 cells is usually associated with the release of IL-12 which is the main IFNγ-inducing cytokine for T cells and NK cells [73] and it has been shown that efficient immune response against *R*. *typhi* in C3H/HeN mice was associated with enhanced serum levels of IL-12 on day 5 post infection [57]. IL-12, however, was neither detectable in the sera of C57BL/6 RAG1^-/-^ nor C57BL/6 wild-type mice at any point in time upon *R*. *typhi* infection [48]. IL-12 is predominantly derived from antigen-presenting cells (APC) such as MΦ and dendritic cells (DCs) and is usually induced by the recognition of bacteria via pattern recognition receptors such as toll-like receptors (TLR) [74]. The analyses of MΦ responses, however, revealed that these cells generally do not react to *R*. *typhi* in a classical manner. Although MΦ up-regulated MHCI and CD80 and showed temporary upregulation of MHCII after infection with *R*. *typhi in vitro*, neither proinflammatory cytokines including IL-12 nor bactericidal NO were released. In contrast to the infection of MΦ *in vitro*, however, infiltrating MΦ in the CNS of *R*. *typhi*-infected C57BL/6 RAG1^-/-^ mice expressed iNOS and, thus, released NO. Whether the activation of APC such as MΦ *in vivo* is directly induced by *R*. *typhi* or supported by other mechanisms such as locally expressed mediators in the affected tissue and how efficient induction of CD4^+^ T_H_1 cells is achieved is unknown.

IFNγ and TNFα produced by CD4^+^ T_H_1 cells [75] are important mediators of protection against intracellular pathogens. IFNγ induces the expression of iNOS and subsequent release of NO in MΦ [70, 76] and endothelial cells [77] both of which are target cells of *Rickettsiae* [4, 78, 79]. Also TNFα can induce iNOS expression in MΦ and synergizes with IFNγ in this effect [80]. In this way, these cytokines support the bactericidal activity of these cells and contribute to bacterial elimination. In concordance, we observed that the addition of IFNγ to *R*. *typhi*-infected bmMΦ *in vitro* leads to reduced bacterial growth and, thus, enhanced bacterial killing although the amount of IFNγ used for these experiments did not induce the release of detectable amounts of NO. Immune CD4^+^ T cells from *R*. *typhi*-infected C57BL/6 mice, however, produced very high amounts of IFNγ in the presence of infected bmMΦ and induced NO release *in vitro*. Furthermore, immune CD4^+^ T cells dramatically reduced bacterial growth in MΦ cultures *in vitro* compared to cultures with control CD4^+^ T cells. This effect was partially inhibited by the neutralization of IFNγ although neutralization of IFNγ was clearly not complete. This observation indicates a dominant role of IFNγ as a mediator of bacterial killing. In addition other factors such as TNFα that was also detectable in cultures of immune CD4^+^ T cells and infected bmMΦ at low amounts may contribute to bacterial elimination.

*In vivo* infiltrating MΦ in the CNS of C57BL/6 RAG1^-/-^ mice colocalized with infiltrating IFNγ-producing CD4^+^ T cells and expressed iNOS at enhanced frequencies. As these infiltrating MΦ were found to harbor the bacteria, this observation suggests that IFNγ-mediated MΦ activation contributes to bacterial elimination and defense against *R*. *typhi in vivo*. Both IFNγ and TNFα have been demonstrated to play an important role in protection against *Rickettsiae*. For example, C3H/HeN mice depleted of either IFNγ or TNFα showed enhanced disease upon infection with a dose of *R*. *conorii* that normally does not result in symptomatic disease [58] and similar observations were made for *R*. *typhi* infection of C3H/HeN mice [57]. Furthermore, a strongly enhanced susceptibility of C57BL/6 IFNγ^-/-^ mice for the infection with *R*. *australis* was observed [55]. Interestingly, both neutralization of IFNγ as well as of TNFα resulted in impaired NO production and overwhelming disease in *R*. *conorii*-infected C3H/HeN mice [58]. Therefore, both cytokines obviously play a role in CD4^+^ T cell-mediated protection by activating MΦ.

Apart from MΦ, immune CD4^+^ T cells also induced iNOS expression in microglia. These cells normally do not express the enzyme in *R*. *typhi* infection and also do not take up the bacteria [48]. Microglial accumulation and activation is associated with neurodegenerative diseases such as multiple sclerosis [81] and Alzheimer´s disease [82]. Activated microglia can directly mediate neuronal damage which is usually associated with iNOS expression [83, 84]. Thus, additional activation of microglia, especially the induction of iNOS in these cells by CD4^+^ T cells, may not directly participate in the elimination of *R*. *typhi* but may have rather non-beneficial immunopathological effects. The observation that the bacteria were not detectable anymore in CD4^+^ T cell recipients that succumbed to the infection, suggests that neurological disease caused by neuronal damage in the CNS of these animals [48] is at least in part an immunopathological effect rather than a result of cellular destruction by *R*. *typhi* itself.

Thinking about vaccination, the induction of an efficient CD8^+^ T cell response is considered the most promising means. Such responses, however, are difficult to induce as antigen must be introduced into the MHCI presentation pathway. Our results, however, show that CD4^+^ T cells can be as protective as CD8^+^ T cells in *R*. *typhi*-infected mice, provided that these cells are present in time. Moreover, CD4^+^ T cells prevented recurrence of disease. These findings suggest that CD4^+^ T cell-inducing vaccination might be as effective as the induction of CD8^+^ T cells. Vaccination with conserved CD4^+^ T cell antigens of TG *Rickettsiae* might even protect against both *R*. *typhi* and *R*. *prowazekii* because it has been shown that animals experimentally infected with *R*. *typhi* are immune to *R*. *prowazekii* infection and vice versa and that similar solid cross-immunity exists for humans [85].

## Supporting Information

S1 Fig CD4^+^ and CD8^+^ T cell-deficient C57BL/6 mice survive the infection with *R*. *typhi* and adoptive transfer of isolated CD4^+^ T cells before day 63 protects C57BL/6 RAG1^-/-^ mice. CD4^+^ T cell-deficient C57BL/6 MHCII^-/-^ and CD8^+^ T cell-deficient C57BL/6 MHCI^-/-^ mice were infected with *R*. *typhi*. None of the animals showed symptoms of disease at any point in time and all mice survived the infection (**A**). Immune CD4^+^ and CD8^+^ T cells were isolated from C57BL/6 mice on day 21 post *R*. *typhi* infection and adoptively transferred into *R*. *typhi*-infected C57BL/6 RAG1^-/-^ mice on day 55 post infection (n = 5 for each group). *R*. *typhi*-infected control animals received PBS instead of T cells (n = 5). All CD4^+^ and CD8^+^ T cell recipients survived the infection (**B**).
(TIF)

S2 Fig *R*. *typhi* is not detectable in the organs of non-infected C57BL/6 RAG1^-/-^ CD4^+^ and CD8^+^ T cell recipients. The bacterial load in the brain, spinal cord, spleen and lung (y-axis) of non-infected control C57BL/6 RAG1^-/-^ mice that received either immune CD4^+^ or CD8^+^ T cells from C57BL/6 mice (x-axis) was determined by qPCR on day 7 post transfer (**A**; n = 2) and on day 210 post infection when the experiments were terminated (**B**; n = 3). *R*. *typhi* was generally not detectable at all in these animals, excluding that contaminating bacteria that might have been present in the T cell preparations contributed to the infection. Furthermore, these mice did not show symptoms of disease at any point in time.
(TIF)
