# Supplementary figures and images for: CD4+ T Cells Are as Protective as CD8+ T Cells against Rickettsia typhi Infection by Activating Macrophage Bactericidal Activity
Source: PLoS Negl Trop Dis. 2016 Nov 22;10(11):e0005089. doi: 10.1371/journal.pntd.0005089 (PMC5119731; doi:10.1371/journal.pntd.0005089)

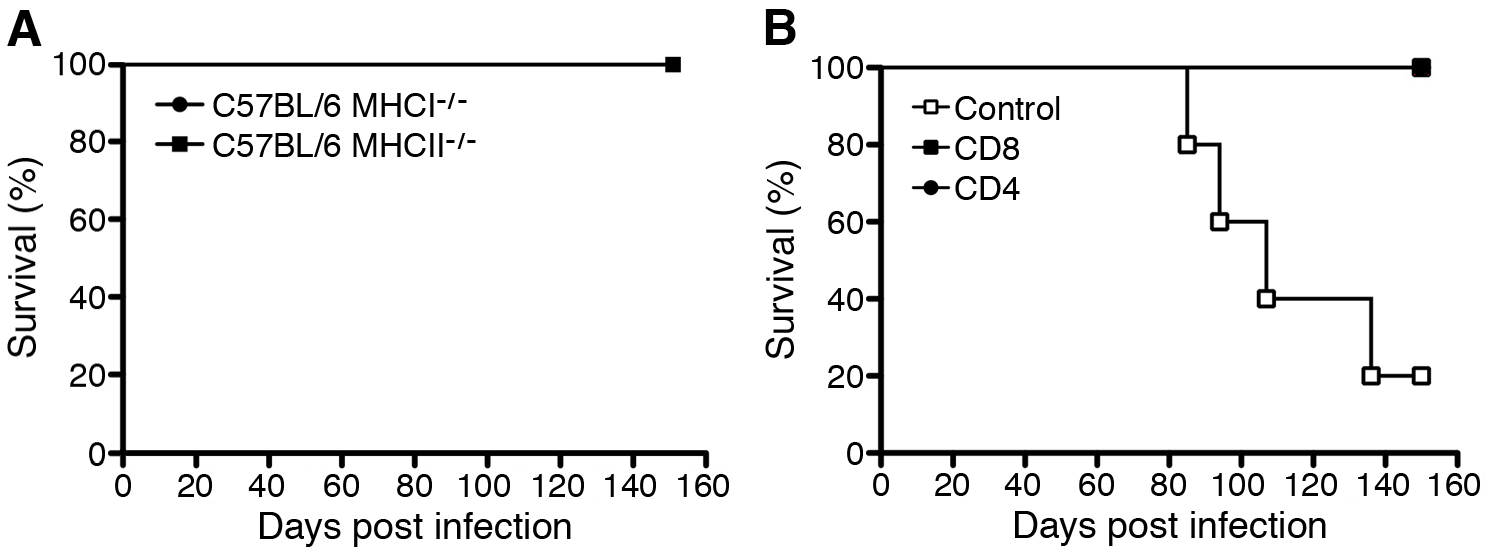

Supplement: S1 Fig — CD4+ T cell-deficient C57BL/6 MHCII-/- and CD8+ T cell-deficient C57BL/6 MHCI-/- mice were infected with R. typhi. None of the animals showed symptoms of disease at any point in time and all mice survived the infection (A). Immune CD4+ and CD8+ T cells were isolated from C57BL/6 mice on day 21 post R. typhi infection and adoptively transferred into R. typhi-infected C57BL/6 RAG1-/- mice on day 55 post infection (n = 5 for each group). R. typhi-infected control animals received PBS instead of T cells (n = 5). All CD4+ and CD8+ T cell recipients survived the infection (B). (TIF) [file pntd.0005089.s001.tif]

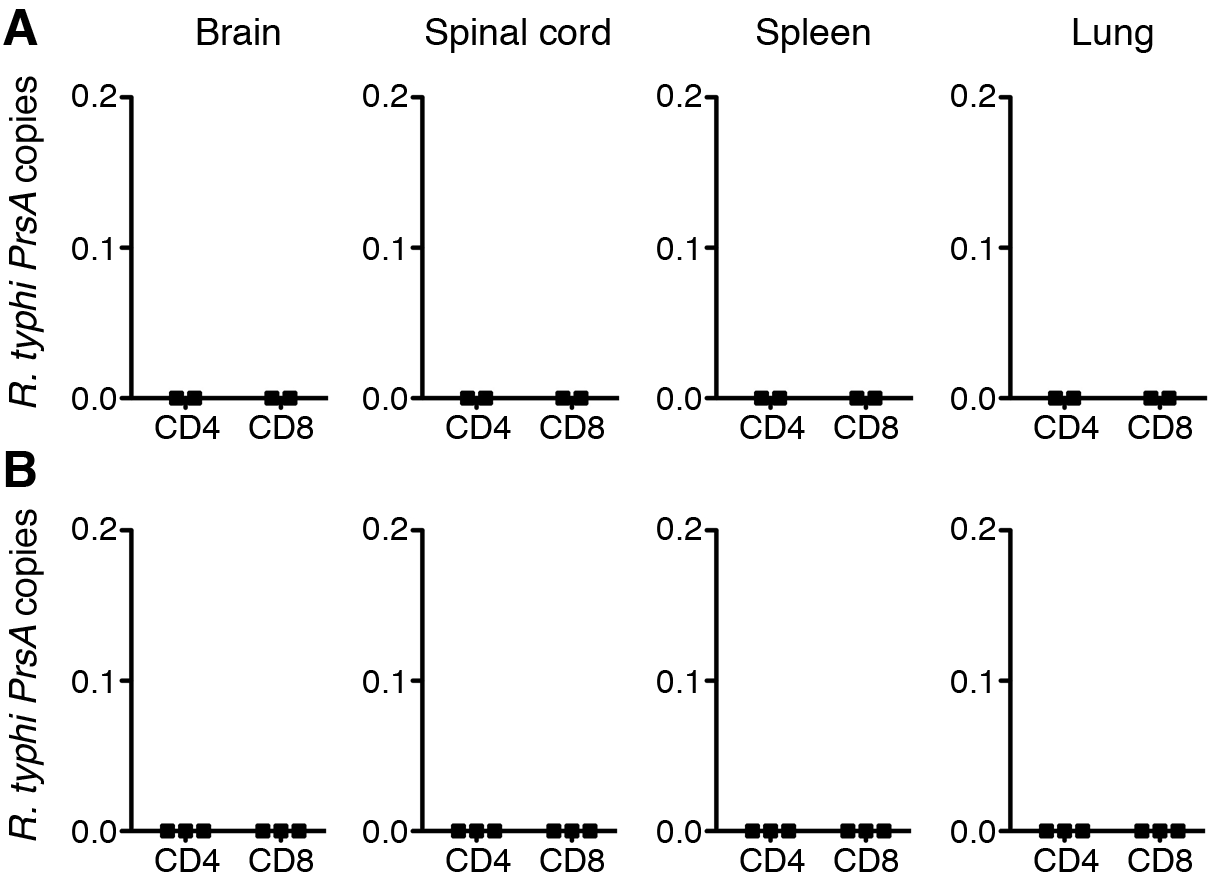

Supplement: S2 Fig — The bacterial load in the brain, spinal cord, spleen and lung (y-axis) of non-infected control C57BL/6 RAG1-/- mice that received either immune CD4+ or CD8+ T cells from C57BL/6 mice (x-axis) was determined by qPCR on day 7 post transfer (A; n = 2) and on day 210 post infection when the experiments were terminated (B; n = 3). R. typhi was generally not detectable at all in these animals, excluding that contaminating bacteria that might have been present in the T cell preparations contributed to the infection. Furthermore, these mice did not show symptoms of disease at any point in time. (TIF) [file pntd.0005089.s002.tif]
